# Levelling the Playing Field: The Role of Workshops to Explore How People With Parkinson's Use Music for Mood and Movement Management as Part of a Patient and Public Involvement Strategy

**DOI:** 10.3389/fresc.2022.873216

**Published:** 2022-07-04

**Authors:** Dawn C. Rose, Ellen Poliakoff, Rebecca Hadley, Ségolène M. R. Guérin, Michelle Phillips, William R. Young

**Affiliations:** ^1^School of Music, Lucerne University of Applied Sciences and Arts, Lucerne, Switzerland; ^2^Division of Neuroscience and Experimental Psychology, The University of Manchester, Manchester, United Kingdom; ^3^University of Hertfordshire, Hatfield, United Kingdom; ^4^Univ. Lille, UMR 9193–SCALab–Sciences Cognitives et Sciences Affectives, Lille, France; ^5^Royal Northern College of Music, Manchester, United Kingdom; ^6^School of Sport and Health Sciences, University of Exeter, Exeter, United Kingdom

**Keywords:** music, rehabilitation, patient and public involvement, interdisciplinary research, Parkinson's disease, patient and public engagement, participatory medicine

## Abstract

From a humanistic perspective, participatory processes in research find support on both ethical and moral grounds. In practical terms however, it is often difficult to establish protocols that best honour (i.e., elicit, capture, and integrate) the opinions of individuals and groups that represent the various specific stakeholders (e.g., from allied health, scientific, and academic disciplines) needed to investigate complex phenomena. Here, we describe a consultation process (funded by Parkinson's UK) devised to explore use of music among people with Parkinson's in relation to potential applications to enhance quality of life. People with Parkinson's were paired with researchers in order to discuss music on an equal footing so as to enable participant empowerment. We describe outcomes that demonstrate avenues of success as a result of this approach and additional insights gained through these processes in the hope of informing future practise. It has been our experience that researchers must establish a balance between (a) ensuring methodological rigour within an appropriate framework, and (b) facilitating informal “playtime” that develops connectivity between participants and enables both creative thinking and reflexive practise amongst stakeholders. We encourage researchers not to underestimate “playtime” as an important vehicle to foster this social interactivity and fuel the good will required to conduct inclusive and relevant research.

## Introduction

The phrase “nothing about me without me” demonstrates a fundamental shift towards empowering patients as co-investigators and shared decision makers, rather than sources of data and recipients of outcomes (1). Although patient and public involvement (PPI) in research has been criticised for being tokenistic or burdensome (pragmatically and/or emotionally), such consultation processes can provide insights that may be overlooked, or at least de-prioritised, by academics and practitioners (2–4). Here we describe a consultation process conducted as part of our ongoing applied approach to arts in health research [as described in the (5) framework]. Specifically, people with Parkinson's (PwP) in our advisory group asked us to investigate which music they should use for a variety of purposes (6, 7). Rather than take a prescriptive approach, we took the opportunity to explore the topic together, considering ways in which PwP currently use music (how, when and why they listen to, use, imagine or remember music). This was planned as a two-way process whereby researchers and PwP worked together to develop insights with the aim of informing a new music-based intervention for PwP and providing resources for their caregivers, medical professionals, and practitioners on which music to choose for which purposes in Parkinson's care.

## Method

### Participants

A network grant from Parkinson's UK enabled a 2 day event that brought 10 PwP and two of their caregivers together with a multidisciplinary team of 12 researchers (music and dance psychologists, biomechanic/kinematic experts, biological, sport- and neuro-scientists).

### Procedure

We prepared a series of playful tasks designed to reduce the power hierarchy that can be felt within PPI in research (2, 8). As everyone is an expert in their own preferred music, we focused on this as a starting point that could “level the playing field” by valuing individual contributions and avoid typical hierarchies associated with expertise *per se* (9, 10). All attendees were provided with an itinerary for the event prior to meeting to ensure consent to participate was informed, to provide a sense of structure to assuage anxiety about what would happen when, and to manage expectations (11). Further rationale for each task and the structure of the 2 days is detailed hereafter.

### Parkinson's UK Network Grant Itinerary

#### Day 1. Welcome Buffet (12:30)

The first day began with a buffet lunch served in the same space as the introductory session for two reasons:

1. To enable personalised greetings and informal *ad-hoc* introductions to develop a sense of social connectedness and enable participants to familiarise themselves with the space.
2. To facilitate staggered arrivals due to range of transport (flights/trains/taxis/personal vehicles) and provide refreshments to alleviate travel fatigue and accommodate the medication regimens of attendees.

#### Day 1. Introductory Session (14:00–17:00)

The first event was designed to try to reduce power hierarchy that can be felt within PPI work in research (2, 8). Attendees introduced themselves and their relationship with music using only one slide. Some attendees took a playful approach, including pictures of themselves, and/or their families, their pets and gardens. One participant, who found public speaking difficult, asked for a short statement about why music was important to her to be read on her behalf:

“I was diagnosed with Young Onset Parkinson's in 2012 at the age of 48. Six and a half years later, I am still not much closer to understanding how Parkinson's works, but what I have experienced is how variable my mobility can be, and how I can change almost instantly, and quite unpredictably, from moving freely to walking extremely slowly and with huge effort. I know from attending Dance for Parkinson's here at the university that there is something about music that makes me move more easily and naturally, and lifts my mood, and whatever that something is, I would like some more of it please! I feel lucky that there are people here investigating the relationship between music and mobility in Parkinson's and I am happy to be involved in studies that further our understanding of how music can be of benefit to us. After all, music is non-invasive, non-addictive, freely available and doesn't wear off with repeated use or have the unwanted side effects of the Parkinson's drugs that we take every day.”

Through previous meetings with our Parkinson's advisory group, we knew that PwP disliked being “lectured at” about Parkinson's. In addition, through our collaborations, we knew that researchers wanted to understand more about what it is like to live with Parkinson's. Therefore, we chose interactive tasks that fostered engagement based on shared experiences about music, and that did not necessarily focus on their symptoms. This playful approach provided a lightness that fostered the feeling that the workshops were safe spaces where each person's voice could be heard and was valued. This approach also facilitated discussions between researchers from different disciplines since they had to avoid field-specific vocabulary that can lead to a reduction in interdisciplinary understanding (12, 13).

#### Day 1. Celebratory Meal (18:00–21:00)

The main meal was arranged on the first rather than last day to accommodate travel arrangements and allow PwP to rest after the workshops. During planning, PwP said they would rather not wait too long to eat after the introductory session. Accordingly, an advance menu selection was arranged. Although seat planning was suggested, attendees preferred to self-select the location of their seats. Though PwP and researchers grouped at opposite ends of the table, this seemed to be a chance to catch up, rather than experienced as partisan.

#### Day 2. Workshop 1 (10:00–12:30)

To accommodate medication and morning routines, day 2 started at 10 am. After a 10 min introduction, each PwP was paired with one of the researchers to consider three related topics. The researchers tended to take the role of recording answers, although they also completed the questions in a separate space (see Supplementary Materials 1).

##### Task 1 (10:20–10:50): Auditory Cueing

This task explored the relationship between sound and movement, reasons to move (i.e., intention), types of sounds that encourage movements, and reasons for listening to and/or using music. Participants made word lists for each of the five questions.

##### Task 2 (11:10–11:40): Name That Tune!

Participants were asked to provide examples of songs they considered to be (a) motivating and (b) relaxing, and to discuss what was important to them (e.g., genre, tempo). Song sharing was encouraged, and a range of spaces provided to facilitate this task (i.e., break out rooms to enable music playing). The session ended with an inspiring group sharing of meaningful songs. For example, PwP shared their experience of diagnosis and how they found solace and/or regained their sense of selves through a particular song.

##### Task 3 (11:50–12:20): Measuring Movements

Attendees produced a w*ish list* and *an issues list* in relation to the ways in which data is collected in research studies and experiments. This was an opportunity for bi-directional learning as PwP and researchers could discuss equipment and questionnaires for example and understand more from each of their perspectives.

Following these tasks, an informal lunch was provided (12:30–14:00; sandwiches and snacks were brought to the research space).

#### Day 2. Workshop 2 (14:00–17:00)

During the afternoon, a researcher-only meeting was conducted to develop collaborations according to available resources. The objectives of this meeting were:

To document a research agenda for future studies on the role of music on mood and movement for PwP.
To identify appropriate empirical measures to evaluate the effect of music on movement and mood for PwP.
To provide a strategy for public engagement focused on music, mood and movement for and with PwP.
To develop collaborative partnerships for grants to facilitate future projects.

## Outcomes

The event resulted in several tangible and prospective outcomes.

Firstly, the qualitative findings from workshops were analysed thematically to create the set of guidelines shown in Table 1. These were used for a study that arose directly from the workshop: PwP wanted to explore the potential impact of drum circle therapy on their wellbeing and asked the researchers to develop new ways of collecting data that would be less arduous for them. Consequently, a feasibility study focused on a single drum circle session in a motion capture laboratory was conducted (14, 15). In order to continue our PPI work, we included an evaluation questionnaire for that study. This feedback/feedforward approach continues to inform our studies. At present we are working with PwP to develop a new approach to quantifying clinical measures, by combining motion capture and gait mat technology, as part of a recently funded PPI project to develop a new music-based intervention for and with PwP^1^.

**Table 1:** The needs and desires of people with Parkinson's for a music-based intervention.

**Movement**	**Mood**
Include a range of movements (functional mobility).	Use music to motivate/energise.
Use a variety of instruments (learn new skills).	Remember (pre diagnosis), enjoy nostalgia.
Learn about rhythms and music around the world.	Express feelings and/or distract from emotions.
Learn how to move in time to music.	Use lyrics to identify with a storey/message/meaning.
Initiate movement/overcome freezing.	To meet people.
Take less time doing empirical measurements.	To help focus and/or escape.
Incorporate periods of rest in activities.	To relax and soothe.

**Figure 1:**
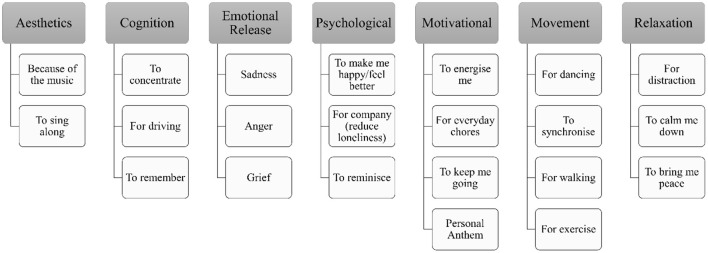
Preliminary categorization of use of music amongst people with Parkinson's in the UK.

Secondly, we were able to formulate seed categories for uses of music among PwP from the workshops (see Figure 1). Using the ideas that emerged in the workshops, we developed an online survey to further investigate how PwP use music in everyday life (16). The survey itself was tested with PwP prior to launch and adjusted according to their feedback. For our recruitment materials, we were able to explain that the survey was developed as a result of direct consultation with PwP who had set the research agenda and helped develop the survey. Whilst we cannot document the impact of this approach in terms of recruitment engagement, we were able to demonstrate authenticity in our commitment to integrating PPI in the research process.

Furthermore, during the survey, we asked PwP to provide examples of songs that they used for particular purposes. This provided a wealth of information which inspired us to develop a new web-based resource, Playlist for Parkinson's. The resource will provide examples of songs and playlists that are actually used by PwP and explain some of the reasons why different types of music may be more or less helpful for different activities (e.g., beat-based music for walking, non-beat-based music for relaxation). We will also supply information useful for practitioners (e.g., tempo) and for researchers (e.g., dynamic musical features) for ecologically valid musical stimuli.

Additionally, at the end of the survey, we asked participants if they would be interested in a new way of disseminating our findings; a concert programmed from examples of which music they use, when and why. The positive response provided a mandate to find funding^2^ to produce two live concerts that will be performed in 2022^3^. Each song will be used to explain some of the psychological reasons aligned to each category of music use (e.g., motivational, anxiolytic), but will also be introduced by a PwP, thereby integrating PPI work into the dissemination process.

Finally, in terms of academic output, the researcher-only meeting led to an open access academic publication on the use of music for movement among PwP (17)^4^ and the studies described above that have been disseminated as conference presentations related to the use of music survey (18, 19). The content of Karageorghis et al. (17) were illustrated as infographics (see Supplementary Materials 2) further extending the value of the collaboration across disciplines and practises.

## Discussion

Although we report a single event herein, we have demonstrated also how this led to the continued integration of PPI processes in our research, putting PwP front and centre in our work. Whilst we acknowledge this is only one approach, it is our opinion that this contributes to best practise in PPI research in several important ways. Firstly, from a pragmatic perspective as discussed by Liabo et al. (10) we learned, for example, to ensure rooms were not only accessible, but that there was enough space to move freely within them, that breaks needed to be 15 rather than 10 min, and that providing transportation (lifts/taxis) enhanced feelings of personal safety and reduced travel fatigue.

Secondly, there was an interpersonal aspect that we found invaluable: by including a variety of short activities, feelings of “camaraderie with purpose” were encouraged. Working in equal partnership has been identified as one of the essential principles of PPI (9, 20). By building equalising tasks into a structured schedule, fears seemed to be disarmed, and mutual respect was encouraged, leading to an amiable approach to teamwork.

These social interactions with PwP had a profound effect on the researchers. Academics are often under pressure to produce impact with their studies, but as Staley (21) describes, impact can be bi-directional. That is, rather than researchers producing results that have impact for the general public, inclusive research processes can have a positive impact on the researchers themselves. As she stated, “changing what researchers ‘think' often informs their research design and practise–it changes what they ‘do”' (p. 158). Informal feedback on the workshops suggested that this was a valuable opportunity to understand much more about how the condition affects PwP and how the use of language (e.g., condition rather than disease) really matters to PwP.

Finally, the feedback from PwP was that meeting such a wide range of researchers who wanted to help them was really interesting and made them feel hopeful and respected. By trying to approach the topic of our research without preconceptions about their experiences, our co-researchers felt listened to and recognised as experts in their own lives.

Nevertheless, future approaches such as this would benefit from collecting basic demographic data to enable better description of the participants for reporting purposes, and more systematic feedback in the form of evaluation from the perspectives of all the participants.

## Conclusion

We used a “light touch” approach that provided a welcoming supportive space that empowered our attendees as co-researchers without placing a burden of research knowledge on them. Although we acknowledge that the topic of music lent itself particularly to playful activities (22), we believe the approach is more widely applicable. Therefore, we encourage others not to underestimate the importance of “playtime” within the processes of participatory research as developing positive feelings and good will fosters longevity in PPI relationships.

## Data Availability Statement

The raw data supporting the conclusions of this article will be made available on request by the authors, without undue reservation.

## Ethics Statement

The Health and Human Sciences Ethics Committee with Delegated Authority [Protocol aLMS/SF/UH/02547(1)] approved a previous study that provided for continued contact with participants who checked a box stating they would like to be a part of further discussions regarding music and Parkinson's. Those who confirmed they would like to continue working on the project were invited to take part in the workshops through the continued contact protocol and were provided with a full itinerary of the event in order to make an informed decision about whether to participate, including being able to ask further questions and knowing they were able to withdraw at any point without any further obligation. All written answers for tasks were anonymised and no recordings were made of the event to protect the identity of the participants.

## Author Contributions

DR created the concept for the workshops and organised and facilitated them with the help of RH who also participated in the workshops. SG and EP also participated in the workshops and, along with WY and MP drafted the manuscript. All authors contributed comments and approved the manuscript.

## Funding

This workshops were funded by a Parkinson's UK Network Grant and paper publication has been financed by the Swiss National Science Foundation Project Grant 10001C_204290. Open access funding was provided by Lucerne University of Applied Sciences and Arts.

## Conflict of Interest

The authors declare that the research was conducted in the absence of any commercial or financial relationships that could be construed as a potential conflict of interest.

## Publisher's Note

All claims expressed in this article are solely those of the authors and do not necessarily represent those of their affiliated organizations, or those of the publisher, the editors and the reviewers. Any product that may be evaluated in this article, or claim that may be made by its manufacturer, is not guaranteed or endorsed by the publisher.

## Footnotes

^1^These projects are partially funded by Parkinson Schweiz and the Swiss National Science Foundation.

^2^Playlist for Parkinson's LIVE is partially funded by the Arnold Bentley New Initiatives Fund held by the Society for Education and Music Psychology Research.

^3^The first at the Royal Northern College of Music (UK, June 2022), and the second at Lucerne University of Applied Sciences and Arts (Switzerland, May 2023).

^4^This was facilitated by a grant from the British Association of Sport and Exercise Scientists.
