# Erratum: Levelling the playing field: The role of workshops to explore how people with Parkinson's use music for mood and movement management as part of a patient and public involvement strategy

**DOI:** 10.3389/fresc.2023.1182198

**Published:** 2023-04-03

**Authors:** Frontiers Production Office

**Affiliations:** Frontiers Media SA, Lausanne, Switzerland

**Keywords:** music, rehabilitation, patient and public involvement, interdisciplinary research, Parkinson’s disease, patient and public engagement, participatory medicine

An Erratum on Levelling the playing field: the role of workshops to explore how people with parkinson’s use music for mood and movement management as part of a patient and public involvement strategy By Rose DC, Poliakoff E, Hadley R, Guérin SMR, Phillips M and Young WR (2022) Front. Rehabilit. Sci. 3:873216. doi: 10.3389/fresc.2022.873216

An omission to the funding section of the original article was made in error. The following sentence has been added: “Open access funding was provided by Lucerne University Of Applied Sciences And Arts”.

The original version of this article has been updated.

