# Supplementary material for: Levelling the Playing Field: The Role of Workshops to Explore How People With Parkinson's Use Music for Mood and Movement Management as Part of a Patient and Public Involvement Strategy
Source: Front Rehabil Sci. 2022 Jul 4;3:873216. doi: 10.3389/fresc.2022.873216 (PMC9397793; doi:10.3389/fresc.2022.873216)
Supplement: Supplementary file 1 [file Data_Sheet_1.docx]

**Supplementary Materials 1: Music, Motivation, Movements and Measures Parkinson's PPI Workshop Tasks**

10:10-10:20 Introduction

10:20-10:50 Task 1: Auditory Cueing?

Q1 - What reasons do you, and/or others have to move (intention)?

Parkinson’s Researchers

Q2 – What types of sounds make you, and/or others, move?

Parkinson’s Researchers

Q3 – Why do you, and/or others, listen to music?

Parkinson’s Researchers

Q4 – Which types of movements do you, and/or others, connect with sounds?

Parkinson’s Researchers

Q5 – What reasons do you, and/or others, use music for?

Parkinson’s Researchers

**11:10-11:40 Task 2: Name That Tune!**

A: Music for Motivation (genre, tempo, mood - what is important? + 12 examples)

Parkinson’s Researchers

B: Music for Relaxation (genre, tempo, mood - what is important? + 12 examples)

Parkinson’s Researchers

**11:50 – 12:20 Task 3: Measuring Movements?**

What would be helpful to people with Parkinson’s?

Wish list ☺

Parkinson’s Researchers

What types of problems might be involved with measuring movements?

Issues List ☹

Parkinson’s Researcher

NOTES:
