# Supplementary figures and images for: Levelling the Playing Field: The Role of Workshops to Explore How People With Parkinson's Use Music for Mood and Movement Management as Part of a Patient and Public Involvement Strategy
Source: Front Rehabil Sci. 2022 Jul 4;3:873216. doi: 10.3389/fresc.2022.873216 (PMC9397793; doi:10.3389/fresc.2022.873216)

**Supplementary Materials 2: Infographics**

**
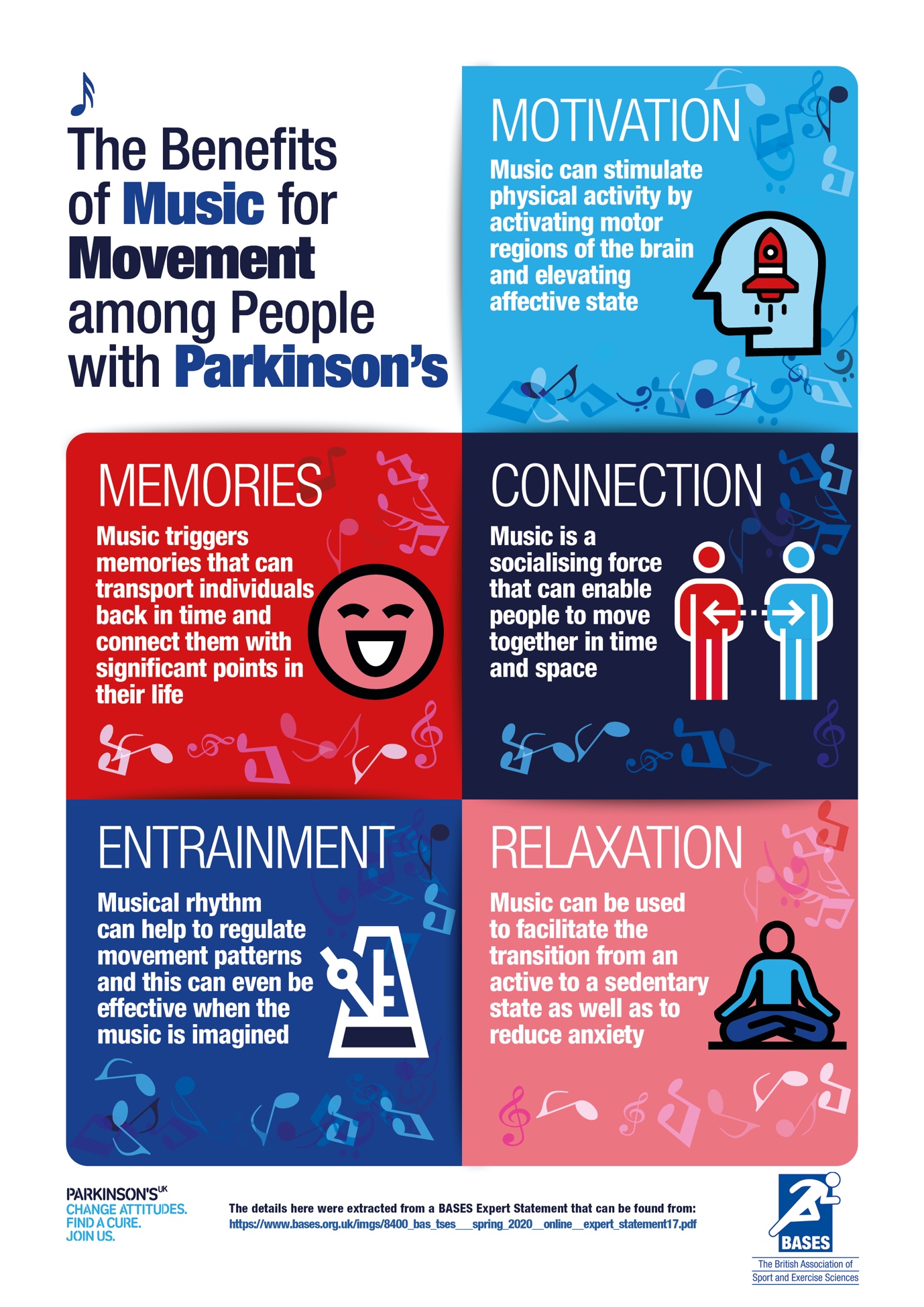
**

**
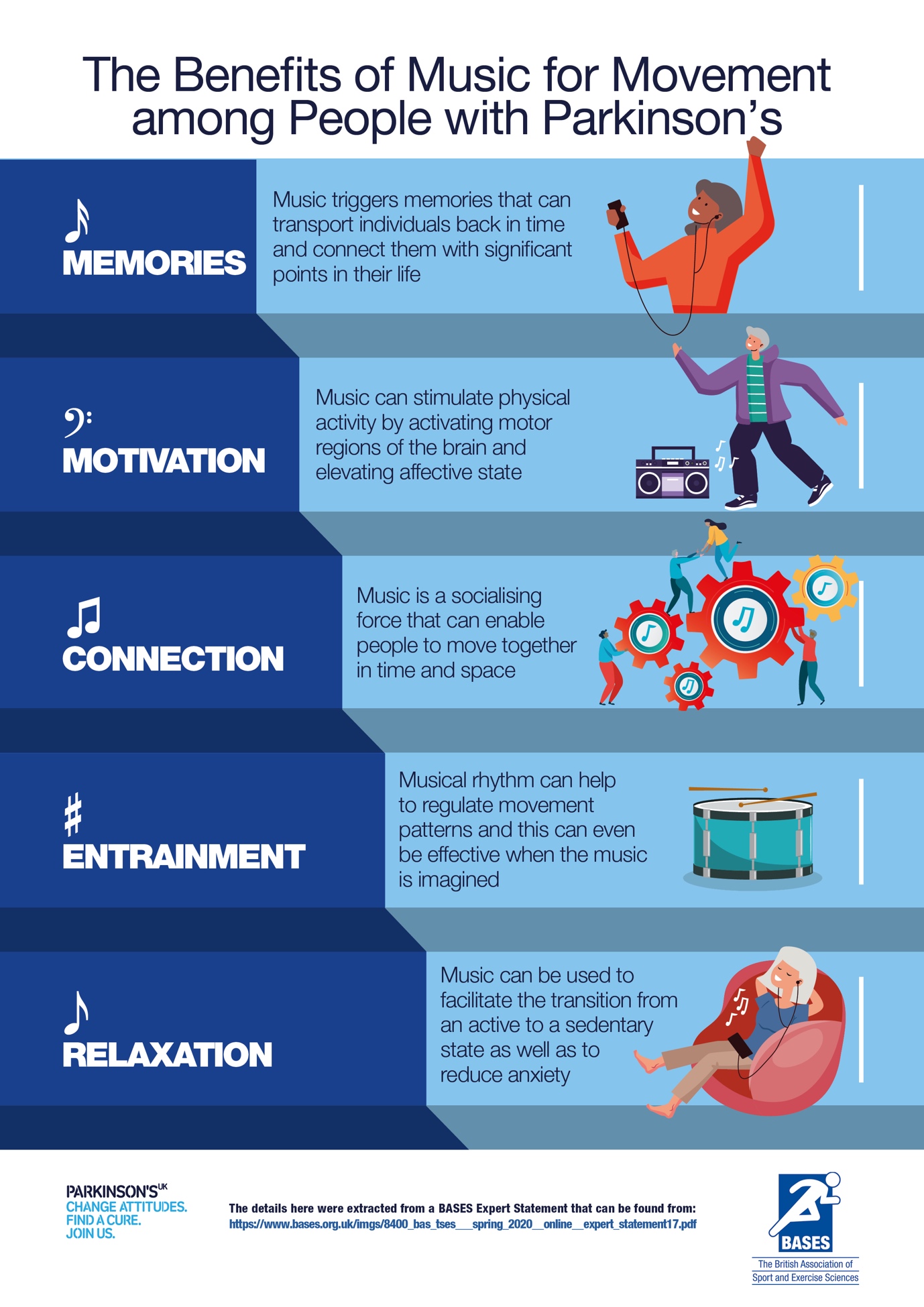
**

**
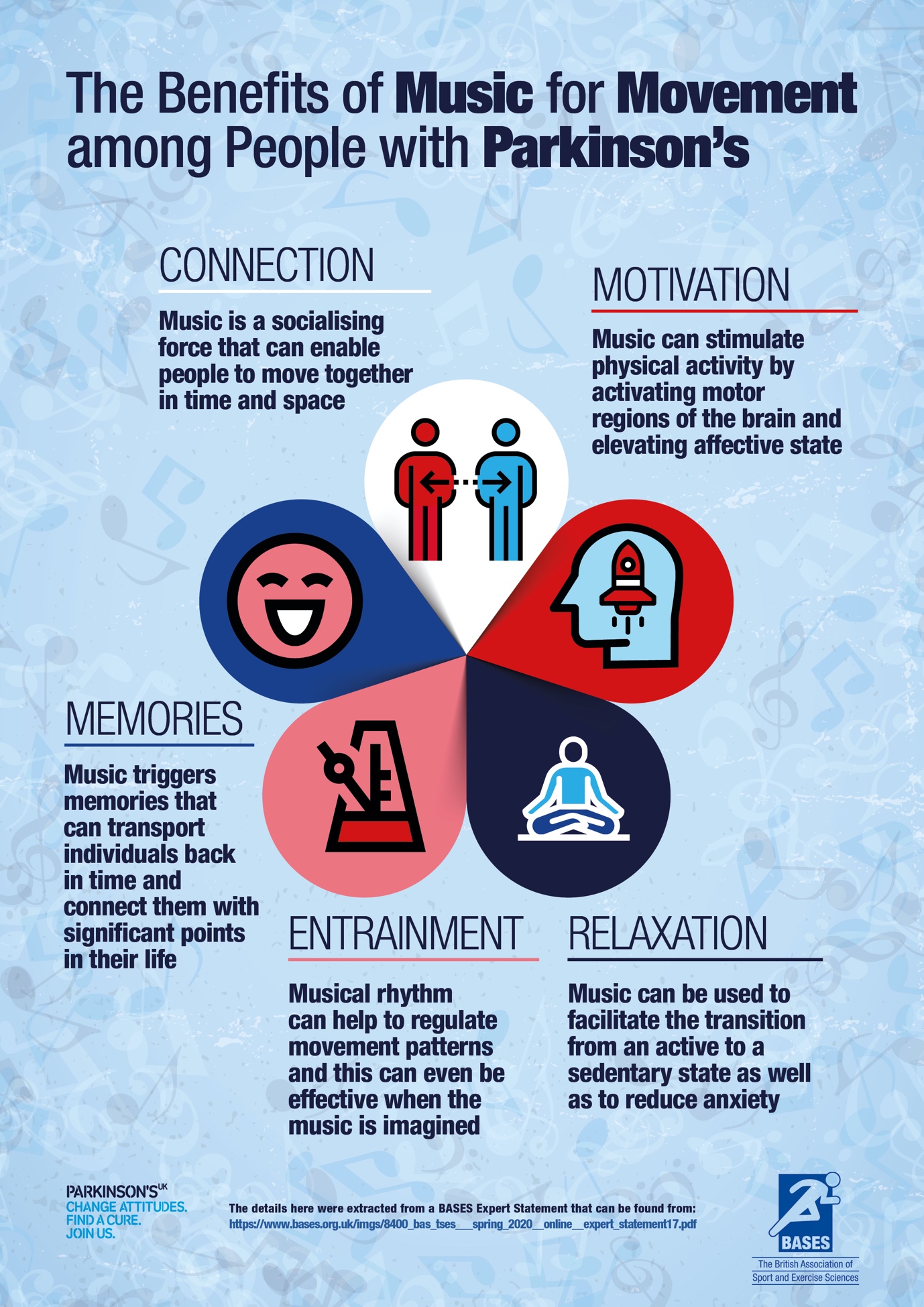
**

**
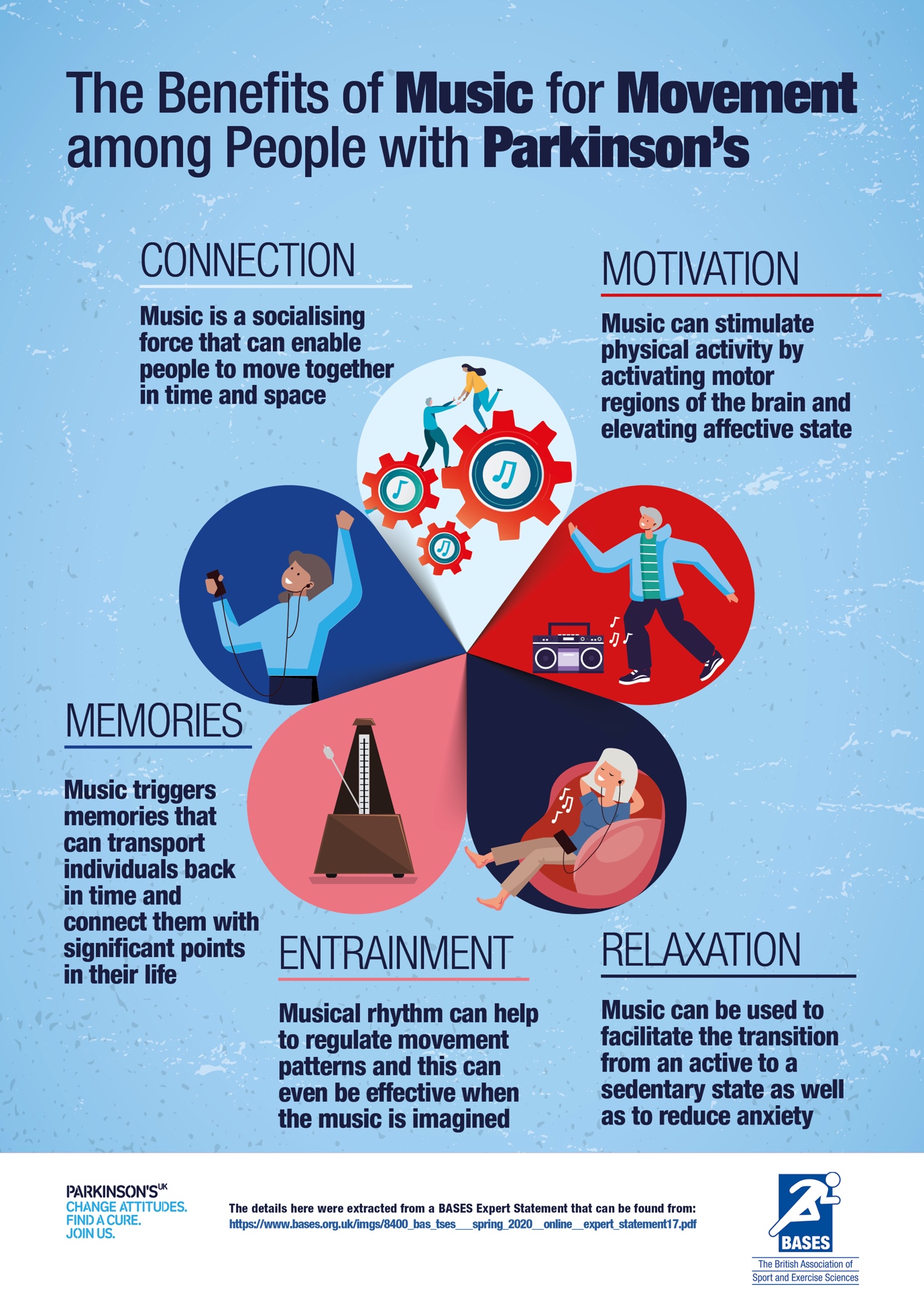
**

Supplement: Supplementary file 2 [file Data_Sheet_2.docx]

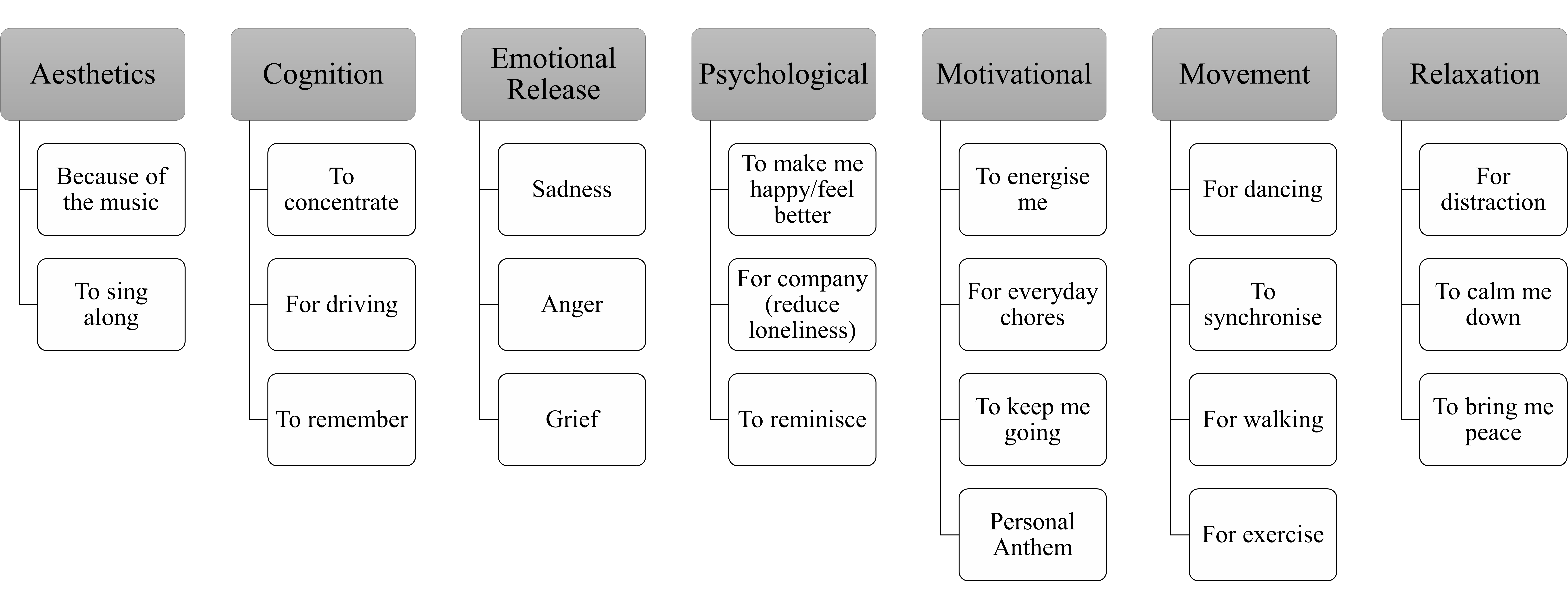

Supplement: Supplementary file 3 [file Image_1.PNG]
